# Comparative Effects of Adapted Taekwondo Versus Tai Chi on Health Status in Independent Older Women: A Randomized Controlled Trial

**DOI:** 10.3390/life15101511

**Published:** 2025-09-25

**Authors:** Tomás Herrera-Valenzuela, Izham Cid-Calfucura, Jordan Hernandez-Martinez, Pablo Valdés-Badilla, José Manuel García-García, Bibiana Calvo-Rico, Cristián Cofre-Bolados, Amaya Pavez-Lizarraga, Verónica Flandes-Vargas, Álvaro Segueida-Lorca, Celso Sánchez-Ramírez

**Affiliations:** 1 Department of Physical Activity, Sports and Health Sciences, Faculty of Medical Sciences, Universidad de Santiago de Chile (USACH), Santiago 8370003, Chile; 2 Department of Physical Activity Sciences, Universidad de Los Lagos, Osorno 5290000, Chile; 3 Departamento de Educación, Facultad de Humanidades, Universidad de la Serena, La Serena 1700000, Chile; 4 Department of Physical Activity Sciences, Faculty of Education Sciences, Universidad Católica del Maule, Talca 3530000, Chile; 5 Sports Coach Career, School of Education, Universidad Viña del Mar, Viña del Mar 2520000, Chile; 6 Facultad de Ciencias del Deporte, Universidad de Castilla-La Mancha (UCLM), 45071 Toledo, Spain; 7 Department of Midwifery, Public Health Center Program, Faculty of Medical Sciences, Universidad de Santiago de Chile (USACH), Santiago 8370003, Chile; 8 Department of Midwifery and Childcare, Faculty of Medical Sciences, Universidad de Santiago de Chile (USACH), Santiago 8370003, Chile

**Keywords:** physical activity, combat sports, older adults, healthy aging

## Abstract

**Background:** Taekwondo (TKD) and Tai Chi (TC) are promising interventions for enhancing health and physical function in older people, yet few studies have compared their effects across multiple domains. This study aimed to compare the effects of TKD versus TC on health status in independent older women. **Methods:** A randomized controlled trial was conducted with two parallel groups: TKD (n = 11) and TC (n = 10). Both groups trained three times per week for 8 weeks. Pre- and post-intervention assessments included anthropometry, submaximal CPX, 2-min step test, Timed Up-and-Go (TUG), isometric mid-thigh pull (IMTP), maximal isometric handgrip strength (MIHS), 30 s chair stand, 30 s arm curl, sit-and-reach, and back scratch. **Results:** Compared with TC, the TKD group showed significantly greater improvements in several cardiorespiratory outcomes, including VO_2_ at VT1 and VT2, power output, VO_2_/HR, OUES, and VE/VCO_2_ slope (*p* < 0.05 to *p* < 0.001; d = 0.69–1.29). TKD participants also exhibited superior gains in maximal and relative IMTP, MIHS, relative MIHS, 30 s arm curl repetitions, and TUG performance (*p* < 0.05 to *p* < 0.001; d = 0.61–1.26). Both groups improved similarly in the 30 s chair stand test (*p* < 0.05). Flexibility outcomes diverged, with TKD improving sit-and-reach and TC showing greater gains in the back scratch test (*p* < 0.05). **Conclusions:** TKD was more effective than TC in improving cardiorespiratory fitness, muscle strength, and balance in older women and may represent a valuable health-oriented training strategy for this population.

## 1. Introduction

Maintaining good health status during aging is essential to prevent functional dependence, a condition particularly prevalent among older women [1]. This dependence negatively affects both physical and mental health, thereby reducing quality of life in later years [2,3]. Among the factors contributing to deterioration is physical inactivity [4], which has been linked to an increased risk of hyperglycemia, hypertension, hypertriglyceridemia, low HDL cholesterol, excess body fat, and a reduction in fat-free mass, muscle strength, balance, and gait speed, particularly in Chilean older women [5]. Conversely, engaging in regular physical activity—whether moderate (≥150 to 300 min/week), vigorous (≥75 to 150 min/week), or a combination of both—has been shown to mitigate many of these risks and promote healthier aging [6]. This contributes to greater functional independence and a better overall health status in older people [7], while also reducing cardiometabolic risk [8], lowering body fat percentage, increasing lean mass, and positively impacting their quality of life [9]. Moreover, it enhances physical performance, which in turn supports autonomy and improves health-related quality of life in this population [10,11]. In this context, the World Health Organization recommends activities such as water-based exercise, strength training, balance, and aerobic exercises for older people [12]. However, other non-traditional approaches—such as Olympic combat sports and martial arts—have also demonstrated positive effects on multiple health outcomes in older adults, including improvements in functional capacity, quality of life, and psychosocial well-being [13,14].

Among the most commonly practiced martial arts in older people are Tai Chi [15] and Taekwondo (TKD) [16]. Tai Chi is a traditional Chinese martial art characterized by slow, continuous, and low-impact movements that emphasize postural control, breathing, and mind–body integration, making it particularly suitable for improving balance and flexibility [15]. In contrast, adapted TKD involves dynamic, structured movements that integrate basic kicks, punches, and steps, thereby providing a more vigorous stimulus to strength, aerobic fitness, and coordination [16]. In a randomized controlled trial (RCT), Valdés-Badilla et al. [17] reported that TKD produced significant improvements in general health (*p* < 0.001), lower-limb muscle strength (chair stand test, *p* = 0.001), aerobic capacity (2-min step test, *p* < 0.001), and flexibility (sit-and-reach, *p* = 0.001) when compared to multicomponent training in older women. Similarly, Elhamrawy et al. [18] found that Tai Chi significantly enhanced upper-limb muscle strength—measured by the 30 s arm curl test and maximal isometric handgrip strength (MIHS)—compared to aerobic training (*p* < 0.05) in older people. Although evidence supporting the individual benefits of TKD and Tai Chi continues to grow, few studies have directly compared their effects across a comprehensive set of physical health indicators. In particular, their comparative impact on morphological variables, cardiorespiratory fitness, balance, muscle strength, and flexibility in independent older women remains largely unexplored.

Therefore, the main aim of this study was to analyze the effects of adapted Taekwondo compared to Tai Chi on key health-related outcomes in older women, including anthropometric indicators, cardiorespiratory fitness, balance, muscle strength, and flexibility. Based on previous evidence [17,18,19], it was hypothesized that an adapted TKD program would lead to greater improvements in cardiorespiratory fitness and muscle strength compared to Tai Chi. In contrast, both interventions were expected to produce similar effects on anthropometric measures, balance, and flexibility.

## 2. Materials and Methods

### 2.1. Study Design

**Figure 1:**
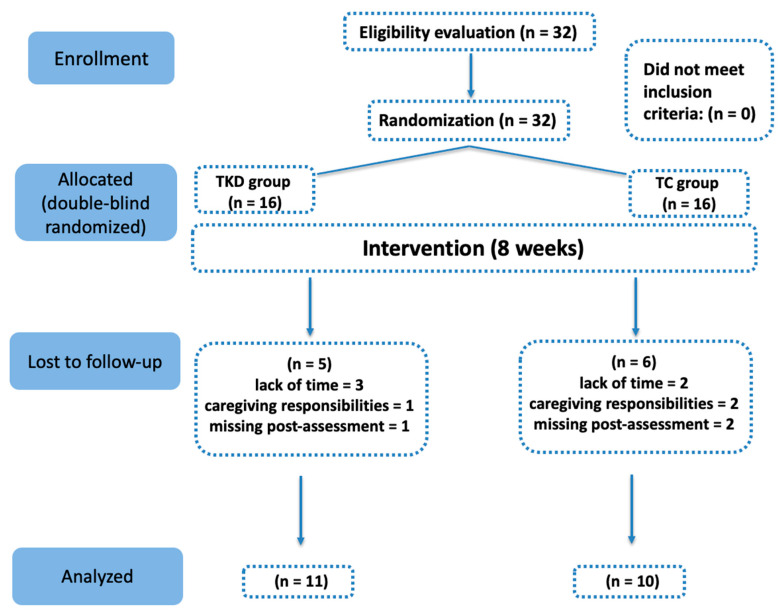
CONSORT flow diagram of participants throughout the study. TKD: Taekwondo; TC: Tai Chi.

The study employed a randomized controlled trial (RCT) design with two parallel groups (TKD and Tai Chi). Due to the nature of the interventions, participants were aware of their group allocation; however, outcome assessors were blinded to group assignment. A repeated measures design was used with a purely quantitative approach. A stratified randomization procedure was applied: prior to allocation, participants were stratified according to baseline cardiorespiratory fitness (VO_2_ at the first ventilatory threshold) and lower-limb strength (isometric mid-thigh pull, IMTP) to ensure a balanced distribution of functional capacity across groups. Randomization was then performed using the Research Randomizer website (https://www.randomizer.org, accessed on 1 March 2025) with a 1:1 allocation ratio. To maintain confidentiality, the CANDIDATE tool was employed to generate anonymous numerical IDs, which were used throughout the study to guarantee traceability. The allocation process was conducted by an independent researcher not involved in the intervention or data analysis, thereby ensuring allocation concealment. Figure 1 presents the flow of participant inclusion.

The intervention lasted 8 weeks (24 sessions), with three 60-min sessions per week on non-consecutive days. The variables assessed included the following: (i) anthropometric parameters (BMI and body weight); (ii) cardiorespiratory fitness indicators (oxygen consumption and power at the first and second ventilatory thresholds [VO_2_VT1, VO_2_VT2], oxygen pulse [VO_2_/HR VT1, VO_2_/HR VT2], respiratory exchange ratio [RER VT1], VE/VCO_2_ slope, oxygen uptake efficiency slope [OUES], cardiorespiratory optimal point [COP], and the 2-min step test); (iii) balance (TUG); (iv) muscle strength (IMTP, MIHS, 30 s chair stand, and 30 s arm curl); and (v) flexibility (sit-and-reach and back scratch tests).

All assessments were performed in the same facilities (sports and community centers) during afternoon hours (2:00–4:00 p.m.), 48 h apart, using identical protocols for pre- and post-intervention testing. Evaluators were blinded to group allocation and remained the same throughout the study. No adverse events or musculoskeletal/cardiopulmonary complications were reported during the intervention.

### 2.2. Participants

The sample size was estimated using G*Power software (version 3.1.9.7, Düsseldorf, Germany), based on a two-tailed *t*-test for independent means. A minimal clinically important difference of 3.46 repetitions in the 30 s chair stand test was considered, with a standard deviation of 3.38 repetitions, according to previous research [20]. Assuming an alpha level of 0.05 and a statistical power (1 − β) of 0.90, the required sample size was 14 participants per group. To account for an anticipated 15% dropout rate, the final recruitment target was increased to 16 participants per group.

Due to a higher-than-expected dropout rate, a post hoc power analysis was conducted using G*Power software. Based on the observed between-group difference in VO_2_ at the first ventilatory threshold (VO_2_ VT1) post-intervention (Cohen’s d = 0.99), with α = 0.05 and sample sizes of 16 (TKD) and 16 (Tai Chi, TC), the achieved power was approximately 77%. Although the initial sample size estimation was based on expected changes in the 30 s chair stand test, the post hoc power analysis was conducted using VO_2_ VT1, one of the variables that showed a significant between-group difference post-intervention, thereby providing a realistic estimate of the statistical power achieved with the final sample.

The following inclusion criteria were applied: (i) women aged 60 to 65 years; (ii) the ability to understand and follow contextualized instructions through simple commands; (iii) functional independence, defined as a score equal to or greater than 43 points on the Preventive Medicine Examination for the Elderly (EMPAM) from the Ministry of Health [21], a national annual preventive health screening tool in Chile that assesses multiple dimensions of elderly functional status including cognitive, sensory, and physical/motor domains, and classifies older adults as “autovalent” (independent), “autovalent with risk”, or “at risk of dependency; and (iv) compliance with at least 85% attendance at the scheduled intervention sessions. The exclusion criteria were as follows: (i) having a disabling disease; (ii) presenting musculoskeletal injuries or undergoing physical rehabilitation that could impair normal physical performance; and (iii) having permanent or temporary contraindications for engaging in physical activity. The age range of 60–65 years was chosen because, in Chile, 60 years is the official threshold for being considered an older adult. Women over 65 were excluded to ensure a relatively homogeneous sample and to reduce variability related to advanced age, comorbidities, and functional decline, which could have affected adherence, safety, and comparability of training responses. A total of 11 older women did not meet the minimum attendance requirement or failed to complete the final assessments: specifically, 5 due to lack of time (n = 3 from the TKD group and n = 2 from the TC group), 3 due to caregiving responsibilities (n = 1 TKD, n = 2 TC), and 3 due to missing post-intervention assessments (n = 1 TKD, n = 2 TC). Thus, 11 participants from the TKD group (mean age: 70.7 ± 2.11 years; body mass: 64.1 ± 11.6 kg; height: 1.58 ± 0.08 m) and 10 from the TC group (mean age: 74.8 ± 6.6 years; body mass: 67.4 ± 8.5 kg; height: 1.59 ± 0.06 m) completed the study. Table 1 presents the participants’ demographic data. Additional details on the baseline group comparisons can be found in the Supplementary Materials (Tables S1–S4). All participants were informed about the purpose and scope of the research and signed an informed consent form authorizing the use of their data for scientific purposes. The study adhered to the ethical principles of the Declaration of Helsinki and was approved by the Institutional Ethics Committee of the University of Santiago de Chile (approval number: N° 392/2024).

### 2.3. Measurements

#### 2.3.1. Anthropometric Variables

Body weight was determined using a mechanical scale (Scale-tronix, Chicago, IL, USA; accuracy to 0.1 kg) while wearing the bare minimum of clothing, and bipedal height was measured using a stadiometer (Seca model 220, SECA, Hamburg, Germany; accuracy to 0.1 cm). The BMI for each older woman was calculated by dividing body weight in kilograms by height in meters squared. Every measurement was conducted in accordance with the guidelines provided by the International Society for the Advances in Kinanthropometry (ISAK) [22].

#### 2.3.2. Cardiorespiratory Fitness

A CPX submaximal test was performed using a Cortex Metamax 3B gas analyzer (Leipzig, Germany), followed by an adapted incremental protocol performed on a Technogym Excite (MR) cycle ergometer [23]. The test started with an initial load of 30 watts, with 10-watt increments every minute, maintaining a cadence between 50 and 60 cycles per minute. The test was stopped after the second ventilatory threshold (VT2), determined according to Wasserman’s proposed graphs number six and nine (ventilatory equivalents and final expiratory pressure of oxygen and carbon dioxide, respectively) [24]. Once the test was stopped, the results were analyzed, and the values measured were categorized into five submaximal parameters: oxygen consumption at the first and second ventilatory thresholds (VO_2_VT1 and VO_2_VT2), power at the first and second ventilatory thresholds, oxygen pulse rate at VO_2_/HR VT1 and VO_2_/HR VT2, respiratory quotient at VT1 (RER), VE/VECO_2_ slope, oxygen consumption efficiency slope (OUES), and cardiorespiratory optimal point (COP) [23].

Additionally, cardiorespiratory fitness was measured using the 2-min step test [25]. Participants were asked to stand erect facing a wall on which the average distance between the patella and the pelvis was marked with colored tape. Participants took steps with their knees raised above the marked point, and the number of repetitions in 2 min was recorded [25].

#### 2.3.3. Balance Performance

Following the recommendations of the fitness test for older people [25], participants were required to rise from a chair with armrests, cross a three-meter walkway, turn around, and return to the chair [25]. Three attempts were made, and the best attempt was recorded in seconds. Two evaluators measured the time using single-beam photocells (Brower Timing System, Draper, UT, USA), and the best of the three attempts was used for statistical analysis.

#### 2.3.4. Muscle Strength Performance

The IMTP was performed using a specialized stand (squat cage) with adjustable height to achieve a correct height position for each participant. The bar was secured in a mid-thigh position with knee and hip angles within 125–145° and 155–165°, respectively [26]. Two force platforms (Pasco PS-2142, PASCO^®^ Scientific, Roseville, CA, USA) recording data at 1000 Hz were used for measurement and analyzed using Pasco Capstone software (version 1.13.4, USA). Older women were instructed to complete two submaximal pulls at approximately 70–80% of their maximum for 6 s with 3 min of recovery between efforts. During the submaximal efforts, corrections were made to the participants’ technique and positioning when required [26]. In addition, participants were asked to report any pain or discomfort during submaximal efforts. All IMTP runs were performed by the same evaluator using the standardized verbal instructions “pull as hard and fast as possible by pushing off the floor” with a “3, 2, 1, go” countdown to increase the reliability of the results [26]. The onset of IMTP was identified using a threshold of 5DE (standard deviations) collected from a 1 s weighing period (standing noise period) prior to the onset of the pull [27]. The best trial of each participant, determined by the highest peak force, was used for statistical analysis. Finally, all runs and submaximal efforts were performed barefoot to reduce the influence of variation in footwear type [26], and no lifting straps were used at any time.

In addition, MIHS was evaluated using a handheld dynamometer (Jamar^®^, PLUS+, Sammons Preston, Patterson Medical, Warrenville, IL, USA) [28]. Participants performed the test seated, with their wrist and forearm in a neutral position, elbow flexed 90°, spine aligned, and shoulders in a neutral position. The dynamometer was set to the first position to promote appropriate contact between the thumb and index finger and ensure an optimal grip. Each participant completed three attempts with their dominant hand after 120 s of rest, and the best value was used for analysis.

Furthermore, the 30 s chair stand test was used to assess lower extremity muscle strength [29]. Participants sat in a chair without armrests, feet flat on the floor, and arms crossed over the chest. Upon the evaluator’s signal, they were instructed to stand up fully and sit back down repeatedly for 30 s. Three trials were performed with 120 s of recovery between each, and the best result was recorded [30].

Lastly, the 30 s arm curl test was administered using a 2.27 kg (5 lb) dumbbell in the dominant hand [25]. While seated with the back straight and feet flat on the floor, participants extended their elbow and then performed full elbow flexion with a supinated forearm, maintaining a neutral wrist position throughout. The evaluator provided a demonstration and allowed two practice trials before the test began. The number of full flexion–extension repetitions completed within 30 s was recorded.

#### 2.3.5. Flexibility Performance

Lower-limb flexibility was assessed using the sit-and-reach test [31]. Participants sat at the edge of a chair positioned against a wall for stability. One foot remained flat on the floor while the opposite leg was extended forward, with the knee fully extended, the heel resting on the ground, and the ankle dorsiflexed at 90°. With one hand placed on top of the other and the middle fingers aligned, participants leaned forward from the hip and attempted to reach their toes, maintaining the position for 2 s. The distance between the fingertips and toes was recorded. A score of zero was assigned if the fingertips touched the toes; a negative value was recorded if the fingers did not reach the toes, and a positive score was noted if the fingertips extended beyond them. Two trials were performed, and the highest score was retained for analysis.

Additionally, upper-limb flexibility was evaluated using the back scratch test [32]. Participants placed one hand behind the head and down the back, with the palm facing the body and fingers pointing downward. Simultaneously, the opposite arm was positioned behind the back with the palm facing outward and the fingers pointing upward, attempting to touch or overlap the middle fingers of both hands. A score of zero was recorded if the fingertips touched. If they did not meet, the gap between fingers was measured (negative score), while overlapping fingers yielded a positive score. Two attempts were conducted, and the best score was used for analysis.

### 2.4. Interventions

Adapted TKD and TC intervention protocols consisted of three 60-min sessions per week, conducted on non-consecutive days over 8 weeks (totaling 24 sessions). The general structure of each session, for both groups, included a 10-min warm-up (joint mobility and low-intensity aerobic exercises), a 40-min main part (adapted TKD or TC), and a 10-min cool-down (dynamic and static flexibility exercises).

The main part of the adapted TKD sessions consisted of non-contact activities, including 10 min of basic stances and specific upper-limb movements (strikes and blocks), followed by 20 min of lower-limb movements (footwork, stances, and kicks), performed both individually and in pairs, with and without equipment (shields and impact pads). The final 10 min involved modified poomsae (a sequence of choreographed arm and leg movements simulating an imaginary combat), adapted to the physical characteristics and capacities of older women [33]. All TKD sessions were led by a certified Taekwondo instructor, recognized by the National TKD Sports Federation (WT), holding a Master’s degree in exercise and health sciences.

The TC sessions followed the standardized 24-form Yang style, practiced for 40 min with slow, coordinated movements, static postures, and breathing and relaxation exercises involving both the upper and lower limbs [34]. All sessions were conducted by a certified Tai Chi master with a Master’s degree in exercise and health sciences.

**Figure 2:**
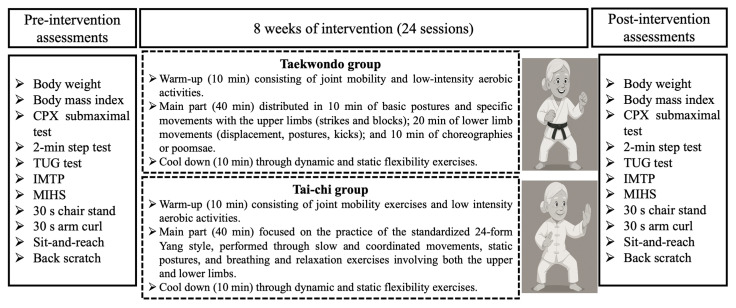
Overview of the training program and assessment timeline. Pre- and post-intervention assessments included anthropometric, cardiorespiratory fitness, balance, muscle strength, and flexibility measures. Both the Taekwondo and Tai Chi groups followed structured training protocols for 8 weeks (3 sessions/week; 60 min per session; 24 sessions total). CPX: cardiopulmonary exercise test. TUG: Timed Up-and-Go. IMTP: isometric mid-thigh pull. MIHS: maximal isometric handgrip strength.

For both intervention groups (TKD and TC), training volume was regulated by controlling work-to-rest ratios, and intensity was adjusted according to prior research recommendations for older people [17]. The perceived exertion was monitored using the Borg Scale (0–10), ensuring values did not exceed 8 points [35]. To ensure participant safety, blood pressure was measured before and after each session, following standardized procedures: seated rest for at least 10 min, back and arms supported, feet flat on the floor, and empty bladder if needed. Post-session measurements were taken to confirm that blood pressure remained stable or close to baseline values [36]. Figure 2 presents a general overview of the training programs and their pre- and post-intervention assessments.

### 2.5. Statistical Analysis

The distribution of the variables was assessed using the Shapiro–Wilk test. Data are presented as mean ± standard deviation (SD). An independent samples *t*-test was used to examine baseline differences between groups. For the TUG test, a repeated measures analysis of covariance (ANCOVA) was conducted, adjusting for pre-test values. For all other outcomes, a two-way repeated measures ANOVA (time × group) was performed, with group (TKD vs. TC) as the between-subjects factor and time (pre- vs. post-intervention) as the within-subjects factor. When significant effects were observed in the ANOVA or ANCOVA, Bonferroni-corrected post hoc tests were conducted. Only post hoc comparisons corresponding to significant interaction effects are reported, as post hoc analyses based solely on main effects were not interpreted. Cohen’s d was calculated to determine the magnitude of between-group differences post-intervention using the formula *d* = (*M*_1_ − *M*_2_)/*SD* [37]. Threshold values were interpreted as follows: <0.20 (trivial), 0.20 (small), 0.60 (moderate), 1.20 (large), 2.00 (very large), and 4.00 (extremely large) [38]. Additionally, partial eta squared (η^2^ₚ) was reported as a measure of effect size for ANOVA/ANCOVA, with values of 0.01 (small), 0.06 (medium), and 0.14 (large) [39]. The significance level was set at *p* < 0.05. All statistical analyses were performed using IBM SPSS Statistics version 30.0 (IBM Corp., Armonk, NY, USA) and GraphPad Prism version 10.0 (GraphPad Software, San Diego, CA, USA), where appropriate.

## 3. Results

### 3.1. Anthropometric Variables

For body weight, no group effect was identified (F_1,19_ = 0.46; *p* = 0.50; η^2^_p_ = 0.02 *small effect*), nor a time effect (F_1,19_ = 0.48; *p* = 0.49; η^2^_p_ = 0.02 *small effect*) and there was no interaction effect (F_1,19_ = 0.25; *p* = 0.61; η^2^_p_ = 0.01 *small effect*), with a small effect size (d = −0.28), indicating a slight reduction in body weight favoring the TC group. Similarly, in BMI, no group effect was identified (F_1,19_ = 0.53; *p* = 0.47; η^2^_p_ = 0.03 *small effect*), nor a time effect (F_1,19_ = 0.46; *p* = 0.50; η^2^_p_ = 0.02 *small effect*) and there was no interaction effect (F_1,19_ = 0.35; *p* = 0.55; η^2^_p_ = 0.02 *small effect*), with a small effect size (d = −0.30), reflecting a slight decrease in BMI favoring the TC group.

### 3.2. Cardiorespiratory Fitness

**Figure 3:**
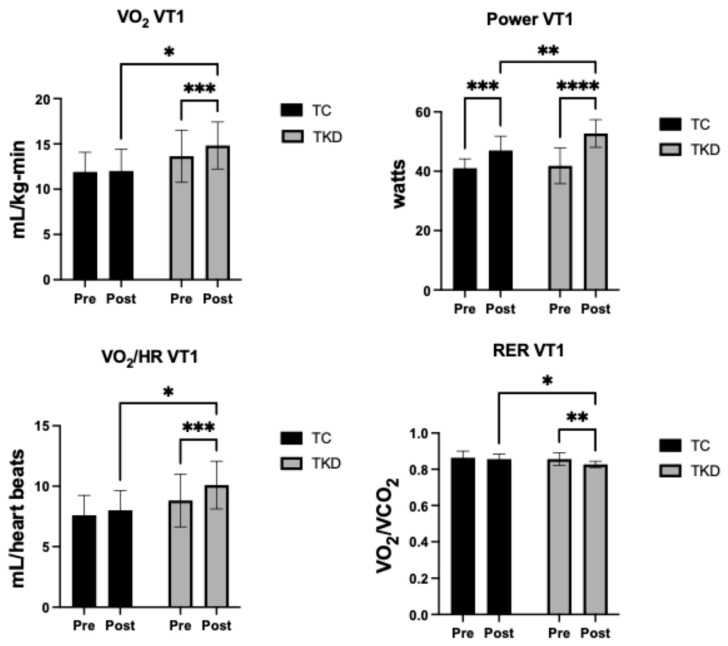
Changes in ventilatory threshold 1 (VT1) cardiorespiratory variables. Pre- and post-intervention values for power output (watts), oxygen consumption (VO_2_ VT1, mL/kg/min), oxygen pulse (VO_2_/HR VT1, mL/heart beat), and respiratory exchange ratio (RER VT1, VO_2_/VCO_2_) in the TKD (Taekwondo) and TC (Tai Chi) groups. Data are presented as mean ± standard deviation. Significant differences were identified between groups and/or time points (* *p* < 0.05, ** *p* < 0.01, *** *p* < 0.001, **** *p* < 0.0001).

For VO_2_ VT1, no group effect was identified (F_1,19_ = 4.32; *p* = 0.05; η^2^_p_ = 0.19 *large effect*). However, a time effect (F_1,19_ = 13.04; *p* = 0.001; η^2^_p_ = 0.41 *large effect*) and an interaction effect (F_1,19_ = 9.29; *p* = 0.006; η^2^_p_ = 0.33 *large effect*) were found. Specifically, significant post-intervention differences were reported between the TKD group and the TC group (*p* = 0.015; 95%CI_dif_ = 0.5719; 5.065), with a large effect size (d = 0.99) in favor of the TKD group. Furthermore, the TKD group improved significantly post-intervention (*p* = 0.001; 95%CI_dif_ = 0.6692; 1.694) in relation to the TC group (*p* = 0.70; 95%CI_dif_ = −0.4376; 0.6376). For power VT1, no group effect was identified (F_1,19_ = 2.97; *p*= 0.10; η^2^_p_ = 0.13 *medium effect*). However, a time effect (F_1,19_ = 85.99; *p* = <0.001; η^2^_p_ = 0.82 *large effect*) and an interaction effect (F_1,19_ = 7.24; *p* = 0.01; η^2^_p_ = 0.28 *large effect*) were found. Specifically, significant post-intervention differences were reported between the TKD group and the TC group (*p* = 0.009; 95%CI_dif_ = 1.466; 9.988), with a large effect size (d = 1.05) in favor of the TKD group. Furthermore, the TKD group improved significantly post-intervention (*p* = <0.001; 95%CI_dif_ = 8.275; 13.54) as did the TC group (*p* = 0.002; 95%CI_dif_ = 3.238; 8.762). For VO_2_/HR VT1, no group effect was identified (F_1,19_ = 4.37; *p* = 0.05; η^2^_p_ = 0.19 *large effect*). However, a time effect was found (F_1,19_ = 13.53; *p* = 0.001; η^2^_p_ = 0.42 *large effect*). No interaction effect was identified (F_1,19_ = 3.68; *p* = 0.07; η^2^_p_ = 0.16 *large effect*). Specifically, significant post-intervention differences were reported between the TKD group and the TC group (*p* = 0.01; 95%CI_dif_ = 0.4241; 3.758), with a large effect size (d = 1.01) in favor of the TKD group. Furthermore, the TKD group improved significantly post-intervention (*p* = 0.007; 95%CI_dif_ = 0.6159; 1.930) in relation to the TC group (*p* = 0.23; 95%CI_dif_ = −0.2889; 1.089). For RER VT1, no group effect was identified (F_1,19_ = 2.52; *p* = 0.12; η^2^_p_ = 0.12 *medium effect*). However, a time effect was found (F_1,19_ = 8.03; *p* = 0.01; η^2^_p_ = 0.30 *large effect*). No interaction effect was identified (F_1,19_ = 2.59; *p* = 0.12; η^2^_p_ = 0.12 *medium effect*). Specifically, significant post-intervention differences were reported between the TKD group and the TC group (*p* = 0.03; 95%CI_dif_ = −0.05540; −0.002055), with a large effect size (d = −1.06), indicating a substantially greater reduction in RER in favor of the TKD group. Furthermore, the TKD group improved significantly post-intervention (*p* = 0.004; 95%CI_dif_ = 0.01019; 0.04800) in relation to the TC group (*p* = 0.40; 95%CI_dif_ = −0.01183; 0.02783). Figure 3 shows the changes in the cardiorespiratory variables at ventilatory threshold 1 (VT1).

**Figure 4:**
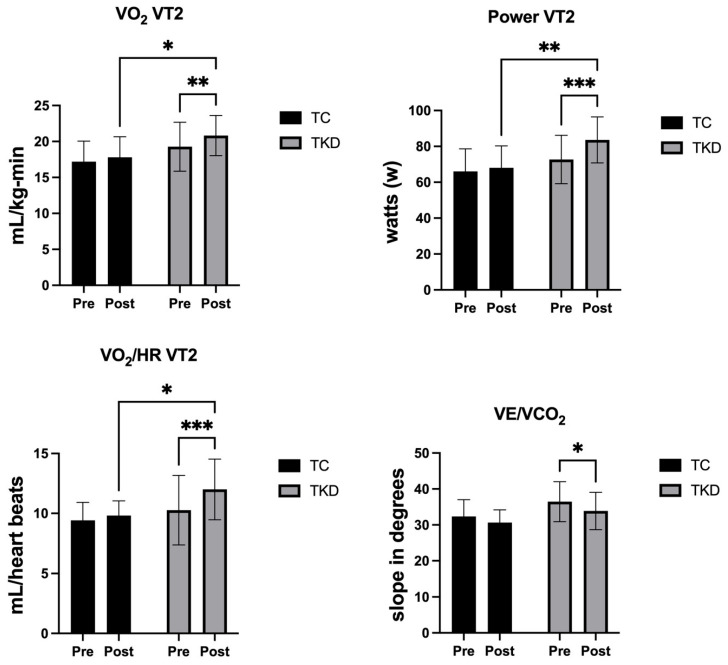
Changes in cardiorespiratory variables at ventilatory threshold 2 (VT2) after 8 weeks of intervention in TKD and TC groups. VO_2_ VT2: oxygen consumption at VT2; Power VT2: power output at VT2; VO_2_/HR VT2: oxygen pulse at VT2; VE/VCO_2_: ventilatory efficiency slope. Data are presented as mean ± standard deviation. TKD: Taekwondo; TC: Tai Chi. Significant differences were identified between groups and/or time points (* *p* < 0.05, ** *p* < 0.01, *** *p* < 0.001).

For VO_2_ VT2, no group effect was identified (F_1,19_ = 3.98; *p* = 0.06; η^2^_p_ = 0.17 *large effect*). However, a time effect was found (F_1,19_ = 13.04; *p* = 0.001; η^2^_p_ = 0.41 *large effect*). No interaction effect was identified (F_1,19_ = 2.53; *p* = 0.12; η^2^_p_ = 0.12 *medium effect*). Specifically, significant post-intervention differences were reported between the TKD group and the TC group (*p* = 0.02; 95%CI_dif_ = 0.3684; 5.668), with a large effect size (d = 0.96), indicating a substantially greater improvement in VO_2_ VT2 in favor of the TKD group. Furthermore, the TKD group improved significantly post-intervention (*p* = 0.001; 95%CI_dif_ = 0.6874; 2.404) in relation to the TC group (*p* = 0.17; 95%CI_dif_ = −0.3000; 1.500). For power VT2, no group effect was identified (F_1,19_ = 4.33; *p* = 0.05; η^2^_p_ = 0.19 *large effect*). However, a time effect (F_1,19_ = 15.78; *p* = 0.008; η^2^_p_ = 0.45 *large effect*) and an interaction effect (F_1,19_ = 7.51; *p* = 0.01; η^2^_p_ = 0.28 *large effect*) were found. Specifically, significant post-intervention differences were reported between the TKD group and the TC group (*p* = 0.008; 95%CI_dif_ = 4.272; 27.00), with a large effect size (d = 1.07), indicating a substantially greater improvement in power VT2 in favor of the TKD group. Furthermore, the TKD group improved significantly post-intervention (*p* = 0.001; 95%CI_dif_ = 6.216; 15.60) in relation to the TC group (*p* = 0.40; 95%CI_dif_ = 2.922; 6.922). For VO_2_/HR VT2, no group effect was identified (F_1,19_ = 2.78; *p* = 0.11; η^2^_p_ = 0.13 *medium effect*). However, a time effect (F_1,19_ = 16.94; *p* = 0.006; η^2^_p_ = 0.47 *large effect*) and an interaction effect (F_1,19_ = 6.59; *p* = 0.01; η^2^_p_ = 0.26 *large effect*) were found. Specifically, significant post-intervention differences were reported between the TKD group and the TC group (*p* = 0.02; 95%CI_dif_ = 0.2633; 4.137), with a large effect size (d = 0.97), indicating a substantially greater improvement in VO_2_/HR VT2 in favor of the TKD group. Furthermore, the TKD group improved significantly post-intervention (*p* = 0.001; 95%CI_dif_ = 0.9808; 2.474) in relation to the TC group (*p* = 0.29; 95%CI_dif_ = 0.3829; 1.183). For VE/VCO_2_, no group effect was identified (F_1,19_ = 3.71; *p* = 0.06; η^2^_p_ = 0.16 *large effect*). However, a time effect was found (F_1,19_ = 5.75; *p* = 0.02; η^2^_p_ = 0.23 *large effect*). No interaction effect was identified (F_1,19_ = 0.24; *p* = 0.62; η^2^_p_ = 0.01 *small effect*). Specifically, the TKD group improved significantly post-intervention (*p* = 0.04; 95%CI_dif_ = −5.180; −0.001544) in relation to the TC group (*p* = 0.20; 95%CI_dif_ = −4.426; 1.006), with a moderate effect size (d = 0.69), indicating a greater reduction in VE/VCO_2_ in favor of the TKD group. Figure 4 shows the changes in the cardiorespiratory variables at ventilatory threshold 2 (VT2).

For COP, no group effect was identified (F_1,19_ = 0.33; *p* = 0.56; η^2^_p_ = 0.02 *small effect*). However, a time effect was found (F_1,19_ = 16.80; *p* = 0.006; η^2^_p_ = 0.47 *large effect*). No interaction effect was identified (F_1,19_ = 1.27; *p* = 0.27; η^2^_p_ = 0.06 *medium effect*). Specifically, the TKD group improved significantly post-intervention (*p* = 0.001; 95%CI_dif_ = −3.994; −1.152) in relation to the TC group (*p* = 0.05; 95%CI_dif_ = −2.950; 0.03044), with a trivial effect size (d = 0.03), suggesting minimal between-group difference despite the significant within-group reduction observed in the TKD group. Finally, for OUES, a group effect was identified (F_1,19_ = 4.39; *p* = 0.04; η^2^_p_ = 0.19 *large effect*). No time effect was found (F_1,19_ = 2.61; *p* = 0.12; η^2^_p_ = 0.12 *medium effect*). However, an interaction effect was found (F_1,19_ = 17.27; *p* = 0.005; η^2^_p_ = 0.48 *large effect*). Specifically, significant post-intervention differences were reported between the TKD group and the TC group (*p* = 0.001; 95%CI_dif_ = 0.04111; 0.1669), with a very large effect size (d = 1.29), indicating a substantially greater improvement in OUES in favor of the TKD group. Furthermore, the TKD group improved significantly post-intervention (*p* = 0.005; 95%CI_dif_ = 0.02952; 0.08867) in relation to the TC group (*p* = 0.09; 95%CI_dif_ = −0.05702; 0.005019).

**Figure 5:**
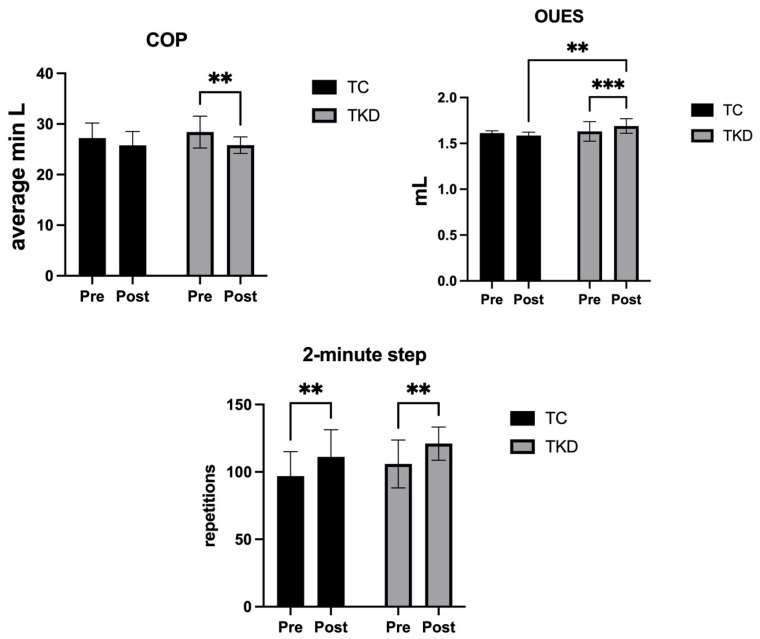
Changes in the cardiorespiratory optimal point (COP), oxygen uptake efficiency slope (OUES), and 2-min step test after 8 weeks of training in the TKD and TC groups. Significant differences were identified between groups and/or time points (** *p* < 0.01, *** *p* < 0.001). TKD: Taekwondo. TC: Tai Chi.

Finally, for the 2-min step test, no group effect was identified (F_1,19_ = 1.92; *p* = 0.18; η^2^_p_ = 0.09 *medium effect*). However, a time effect was found (F_1,19_ = 20.32; *p* = 0.002; η^2^_p_ = 0.52 *large effect*). No interaction effect was identified (F_1,19_ = 0.01; *p* = 0.90; η^2^_p_ = 0.00 *small effect*). Specifically, the TKD group improved significantly post-intervention (*p* = 0.003; 95%CI_dif_ = 5.674; 24.51) as did the TC group (*p* = 0.006; 95%CI_dif_ = 4.423; 24.18) with a moderate effect size (d = 0.58), indicating a slightly greater improvement in the TKD group. Figure 5 shows the changes in the COP, OUES, and 2-min step test after training in the TKD and TC groups.

### 3.3. Balance Performance

For the TUG test, the ANCOVA analysis adjusted for pre-test values identified an interaction effect (F_2,18_ = 52.2; *p* = <0.001; η^2^_p_ = 0.85 *large effect*). Specifically, significant post-intervention differences were reported between the TKD group and the TC group (*p* = 0.04; 95%CI_dif_ = −1.082; −0.005), with a very large effect size (d = −1.26), indicating a substantially greater reduction in completion time in favor of the TKD group.

### 3.4. Muscle Strength Performance

For IMTP, no group effect was identified (F_1,19_ = 0.26; *p* = 0.61; η^2^_p_ = 0.01 *small effect*). However, a time effect (F_1,19_ = 6.54; *p* = 0.01; η^2^_p_ = 0.26 *large effect*) and an interaction effect (F_1,19_ = 10.88; *p* = 0.003; η^2^_p_ = 0.36 *large effect*) were found. Specifically, the TKD group improved significantly post-intervention (*p* = 0.004; 95%CI_dif_ = 83.30; 245.4) compared to the TC group (*p* = 0.61; 95%CI_dif_ = −105.8; 64.23), with a moderate-to-large effect size (d = 0.71), indicating a greater strength gain in the TKD group. Similarly, no group effect was identified for the relative IMTP (F_1,19_ = 0.94; *p* = 0.34; η^2^_p_ = 0.05 *small effect*). However, a time effect (F_1,19_ = 11.95; *p* = 0.002; η^2^_p_ = 0.39 *large effect*) and an interaction effect (F_1,19_ = 14.23; *p* = 0.001; η^2^_p_ = 0.43 *large effect*) were found. Specifically, the TKD group improved significantly post-intervention (*p* < 0.001; 95%CI_dif_ = 1.239; 2.888) compared to the TC group (*p* = 0.82; 95%CI_dif_ = −0.9548; 0.7748), with a large effect size (d = 0.79), indicating a greater improvement in relative strength in the TKD group.

Regarding MIHS-dominant hand, no group effect was identified (F_1,19_ = 1.24; *p* = 0.27; η^2^_p_ = 0.06 *medium effect*). However, a time effect (F_1,19_ = 33.11; *p* = <0.001; η^2^_p_ = 0.64 *large effect*) and an interaction effect (F_1,19_ = 15.77; *p* = 0.008; η^2^_p_ = 0.45 *large effect*) were found. Specifically, the TKD group improved significantly post-intervention (*p* = <0.001; 95%CI_dif_ = 1.534; 2.830) compared to the TC group (*p* = 0.23; 95%CI_dif_ = −0.2797; 1.080), with a moderate effect size (d = 0.61), indicating a greater increase in MIHS in the TKD group. For relative MIHS-dominant hand, no group effect was identified ((F_1,19_ = 1.62; *p* = 0.21; η^2^_p_ = 0.08 *medium effect*), nor a time effect (F_1,19_ = 0.84; *p* = 0.36; η^2^_p_ = 0.04 *small effect*) or an interaction effect (F_1,19_ = 4.12; *p* = 0.05; η^2^_p_ = 0.18 *large effect*). A significant improvement post-intervention was only reported in the TKD group (*p* = 0.04; 95%CI_dif_ = 0.0007522; 0.06834) in relation to the TC group (*p* = 0.45; 95%CI_dif_ = −0.04844; 0.02244), with a large effect size (d = 0.79), indicating a greater improvement in relative MIHS in the TKD group.

For the 30 s chair stand test, no group effect was identified (F_1,19_ = 4.30; *p* = 0.05; η^2^_p_ = 0.18 *large effect*). However, a time effect was found (F_1,19_ = 13.53; *p* = 0.001; η^2^_p_ = 0.42 *large effect*). No interaction effect was identified (F_1,19_ = 0.02; *p* = 0.98; η^2^_p_ = 0.00 *small effect*). Specifically, the TKD group improved significantly post-intervention (*p* = 0.01; 95%CI_dif_ = 0.4612; 3.902) as did the TC group (*p* = 0.01; 95%CI_dif_ = 0.3954; 4.005), with a large effect size (d = 0.83), indicating a greater overall improvement in the TKD group.

**Figure 6:**
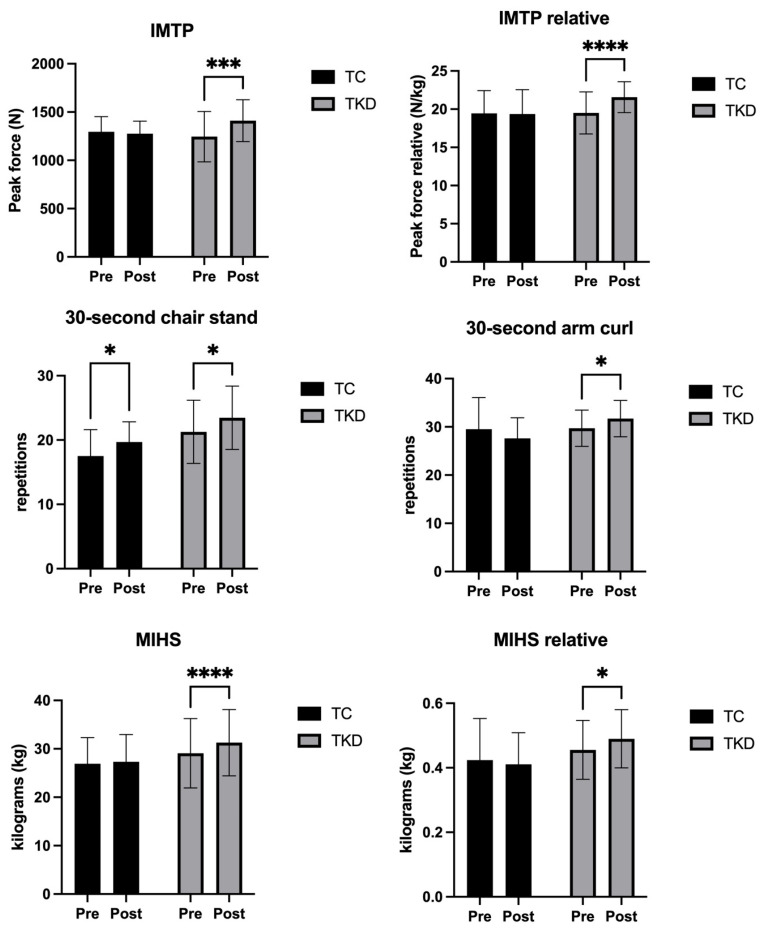
Changes in muscle strength variables (IMTP, relative IMTP, MIHS [dominant hand], relative MIHS [dominant hand], 30 s chair stand, and 30 s arm curl) after 8 weeks of training in the TKD and TC groups. * Significant differences were identified between groups and/or time points (* *p* < 0.05, *** *p* < 0.001, **** *p* < 0.0001). TKD: Taekwondo. TC: Tai Chi. IMTP: isometric mid-thigh pull. MIHS: maximal isometric handgrip strength.

Finally, in the 30 s arm curl test, no group effect was identified (F_1,19_ = 1.25; *p* = 0.27; η^2^_p_ = 0.06 *medium effect*) nor was a time effect found (F_1,19_ = 0.05; *p* = 0.94; η^2^_p_ = 0.00 *small effect*). However, an interaction effect was identified (F_1,19_ = 8.55; *p* = 0.008; η^2^_p_ = 0.31 *large effect*). Specifically, the TKD group improved significantly post-intervention (*p* = 0.04; 95%CI_dif_ = 0.07440; 3.926) compared to the TC group (*p* = 0.06; 95%CI_dif_ = −3.920; 0.1196), with a large effect size (d = 0.93), indicating a substantially greater improvement in upper-limb muscle endurance in the TKD group. Figure 6 shows the changes in muscle strength performance variables following the 8-week intervention in the TKD and TC groups.

### 3.5. Flexibility Performance

For the sit-and-reach test, no group effect was identified (F_1,19_ = 0.33; *p* = 0.57; η^2^p = 0.02, *small effect*). However, a significant time effect was found (F_1,19_ = 10.12; *p* = 0.004; η^2^p = 0.35, *large effect*), with no interaction effect (F_1,19_ = 0.02; *p* = 0.87; η^2^p = 0.00, *small effect*). Specifically, the TKD group improved significantly in sit-and-reach performance post-intervention (*p* = 0.02; 95% CI diff = −4.745; −0.3458), while the TC group showed only a borderline change (*p* = 0.05; 95% CI diff = −4.607; 0.006965), with a small effect size (d = 0.24). This pattern suggests a modest advantage for the TKD group in enhancing lower-limb flexibility.

**Figure 7:**
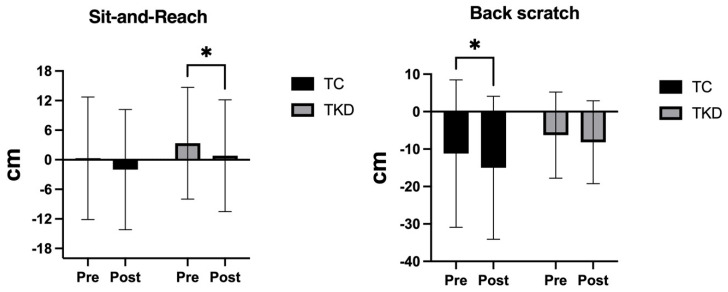
Changes in sit-and-reach and back scratch flexibility tests after 8 weeks of training in the TKD and TC groups. * Significant differences were identified between time points (* *p* < 0.05). TKD: Taekwondo. TC: Tai Chi.

Conversely, for the back scratch test, no group effect was identified (F_1,19_ = 0.76; *p* = 0.39; η^2^p = 0.03, *small effect*). A significant time effect was found (F_1,19_ = 5.66; *p* = 0.02; η^2^p = 0.23, *large effect*), with no interaction effect (F_1,19_ = 0.62; *p* = 0.44; η^2^p = 0.03, *small effect*). Specifically, the TC group improved significantly in back scratch performance post-intervention (*p* = 0.04; 95% CI diff = −7.433; −0.1668), whereas the TKD group showed no meaningful change (*p* = 0.26; 95% CI diff = −5.373; 1.555), with a small-to-moderate effect size (d = 0.44). This finding suggests that TC provided a more effective stimulus for enhancing upper-limb flexibility. Figure 7 shows the changes in flexibility performance variables following the 8-week intervention in the TKD and TC groups.

## 4. Discussion

This study aimed to analyze the effects of TKD versus TC programs on anthropometric variables (body weight, BMI); cardiorespiratory fitness variables (CPX submaximal test and 2-min step test); balance variables (TUG); muscle strength variables (IMTP, MIHS, 30 s chair stand, 30 s arm curl) and flexibility variables (sit-and-reach and back scratch). We hypothesized that an adapted TKD program would produce greater improvements in cardiorespiratory and muscle strength variables compared to TC training. Furthermore, both modalities were expected to produce similar improvements in anthropometric measures, balance, and flexibility. However, our findings show that this hypothesis was only partially fulfilled.

Specifically, the results of our study were as follows: (i) No significant changes in BMI or body weight were observed in either group. (ii) Post-intervention comparisons favored the TKD group over the TC group for several cardiorespiratory variables, including VO_2_VT1, VO_2_/HR VT1, RER VT1, VO_2_VT2, power VT2, VO_2_/HR VT2, and OUES. Additionally, TKD showed significant within-group improvements in VE/VCO_2_ and COP, although between-group differences were not statistically significant. For power at VT1, both groups improved, but the difference favored TKD. (iii) The 2-min step test improved significantly in both groups post-intervention. (iv) Regarding balance, the TKD group outperformed the TC group in the TUG test. (v) In muscle strength, significant improvements favoring the TKD group were observed for IMTP, relative IMTP, MIHS, relative MIHS, and the 30 s arm curl. For the 30 s chair stand, both groups showed significant gains. (vi) Finally, for flexibility, a significant decline was observed in the TKD group for the sit-and-reach test, while the TC group showed a decline in the back scratch test.

### 4.1. Anthropometric Variables

No significant intragroup or intergroup changes in favor of the TKD and TC intervention groups were reported for body weight and BMI. Similarly, Valdés-Badilla et al. [17] did not find significant improvements in the BMI (*p* = 0.07), body fat percentage (*p* = 0.628), and fat-free mass (*p* = 0.317) after TKD and multicomponent training (MCT) interventions in older women lasting 8 weeks through three weekly sessions of 60 min at intensities between 50 and 70% HRmax. This is contrary to what was reported by Siu et al. [40], with TC and conventional exercise interventions for 38 weeks in middle-aged and older people, who reported significant improvements for TC in waist circumference (*p* < 0.001) and body weight (*p* < 0.001) compared to the control condition. As mentioned in previous research [17,41,42], nutritional control has an important role in the effects of exercise through combat sports on body composition in older people. It has been suggested that physical activity plus the implementation of a nutritional plan with protein intake (1.0 to 1.2 g/kg of body weight/day) can improve body composition in older people [20,43]. In this context, our study participants did not follow a nutritional guideline that complemented the TKD and TC interventions, which may have influenced our findings. Considering that dietary habits can be modified [43], it is essential to promote nutritional education in older people through the intervention of a team of nutrition specialists. Conversely, our results for the TC intervention are different from those reported by Siu et al. [40], who reported significant reductions in waist circumference and body weight. However, this may be attributed to the longer duration (38 weeks) compared to our intervention (8 weeks), since long-term interventions have been reported to improve physiological adaptations in lipid metabolism and body composition [44].

### 4.2. Cardiorespiratory Fitness

In the CPX test, significant improvements and differences favoring the TKD group compared to the TC group were identified for VO_2_ VT1, VO_2_/HR VT1, RER VT1, VO_2_ VT2, VT2 power, VO_2_/HR VT2, and OUES. Significant improvements were reported only in the TKD group for VE/VCO_2_ and COP. Regarding VT1 power, both groups showed significant improvements after the intervention; however, the differences between groups favored the TKD group. To our knowledge, this is the first study to analyze the cardiorespiratory responses of TKD versus TC in older women. Our results are similar to those reported by Cofre-Bolados et al. [45], who found significant improvements in VO_2_ VT1, VE/VCO_2_, OUES, and ∆VO_2_/HR after 14 weeks of aerobic and high-intensity interval training (HIIT) in older people. Similarly, previous studies [46] have reported that a 4-week HIIT program increased VO2 peak (9.6%) and anaerobic threshold (13%) in healthy older people. The significant improvements in favor of the TKD group in our study may be attributed to the high-speed intermittent actions performed by the older people (for example, upper-limb strikes and blocks, movements, postures, kicks, and choreographies that simulate an imaginary combat), which may have generated greater cardiorespiratory and neuromuscular stress compared to the TC group that performed slow and controlled full-body movements [47,48]. In this context, the TKD intervention resembles HIIT training, which could have favored better adaptations at the level of oxygen transport and utilization, as well as greater efficiency in gas exchange [49,50]. This interpretation is consistent with recent systematic reviews showing that HIIT induces significant improvements in VO_2_ peak and ventilatory thresholds in older adults, reinforcing its potential as an effective strategy for enhancing CRF in this population [49,50]. Conversely, the TC group only showed improvements in VT1 power. In this sense, the slow and controlled techniques performed in the 24-form Yang-style activities may have been insufficient to improve the cardiorespiratory component [51]. However, they may have led to peripheral muscle improvements in the lower limb, given the significant improvements identified in the 30 s chair stand test, which could have facilitated greater mechanical work in VT1.

On other hand, significant improvements were found for both the TKD and TC groups with no significant differences between groups. This is consistent with the findings reported by Valdes-Badilla et al. [17], who reported significant improvements in the 2-min step test (*p* = 0.004) after an 8-week TKD intervention versus MCT in apparently healthy older women. Similarly, Taylor-Piliae et al. [52] reported significant improvements in the 2-min step test (*p* < 0.001) after a 12-week TC intervention in older stroke survivors. This is in contrast to the findings of Vasquez-Carrasco et al. [42] in apparently healthy older women who, after an 8-week intervention with TKD, boxing (BOX), and elastic band training (EBT) groups, did not present significant improvements for the 2-min step test (*p* = 0.166). This is similar to the findings reported by Valdés Badilla, et al. [33], where no improvements were identified in the 2-min step test (*p* = 0.737) in older women after 16 weeks of TKD training. It has been reported that improvements in performance on tests related to lower-limb muscle strength can improve performance in the 2-min step test due to greater efficiency in the extensor muscles of older individuals [53]. In this context, both groups showed significant improvements in the 30 s chair stand test, contrary to the results of Vasquez-Carrasco et al. [42], which may have favored the finding of significant improvements in the 2-min step test. Furthermore, the improvement in VT1 power in the TKD and TC groups may have contributed to the efficiency of the lower limbs during this test.

### 4.3. Balance Performance

Regarding balance, a significant improvement was identified for the TKD group, while no improvements were found for the TC group. This is in line with the findings of Valdes-Badilla et al. [33], who observed significant improvements in TUG (*p* < 0.001) in apparently healthy older women following a 16-week TKD intervention. It is also similar to the findings reported by Vasquez-Carrasco et al. [42] that apparently healthy older women identified significant improvements in TUG (*p* < 0.001) after an 8-week intervention with TKD, BOX, and EBT groups. Conversely, our findings are different from those reported by Cui et al. [19] in a systematic review with meta-analysis that investigated the effects of TC on balance in healthy older people, where there were significant improvements in the TUG test compared to control conditions (*p* < 0.001). Evidence has shown that TKD and TC can improve TUG performance through increased neuromuscular control of the lower limbs in older people [19,33]. However, significant improvements were only observed in favor of TKD. This may be attributed to the specific characteristics of the techniques used during the intervention. For instance, TC involved slow movements and controlled shifts of body weight forward, backward, laterally, and diagonally, mimicking everyday activities [14]. This may have primarily enhanced static balance and movement control in a controlled setting. However, the TUG test requires dynamic balance and lower-limb speed, which may have been favorable for the TKD group given the punching, kicking, and moving actions performed at the participants’ maximum possible speed [41]. In this context, TKD may have generated greater lower-limb efficiency during gait [54]. Additionally, according to the meta-analysis by Cui et al. [19], TC interventions may require durations longer than eight weeks to produce significant improvements in TUG performance.

### 4.4. Muscle Strength Performance

Significant improvements in IMTP and relative IMTP were found in favor of TKD, while no improvements were reported for the TC group. To our knowledge, this is the first investigation to incorporate IMTP as an outcome measure in older people. However, evidence suggests that combat sport interventions can lead to improvements in maximal isometric lower-limb muscle strength in older people [55,56]. Kujach et al. [55] reported significant improvements in isometric strength during knee flexion and extension after 12 weeks of judo training in older people. This is consistent with the findings of Penn et al. [56], who reported significant improvements in isometric strength of the hip flexors, hip extensors, hip abductors, hip adductors, knee extensors, knee flexors, ankle dorsiflexors, and ankle plantar flexors following eight weeks of individualized TC intervention in older people. Our results reported that TKD significantly improved maximal and relative isometric strength during IMTP. These effects may be attributed to neuromuscular adaptations induced by TKD practice [41], specifically, improved recruitment of high-threshold motor units and reduced coactivation of antagonist muscles [57]. Such adaptations may be mediated by the nature of TKD techniques, which involve rapid punching, kicking, and multidirectional movements. In contrast, the actions performed by the TC group are characterized by smooth, coordinated movements with limited muscular loading [33]. In this context, the discrepancies between our findings for TC and those reported by Penn et al. [56] may be attributed to the individualization of the training applied in their study. Penn et al. [56] categorized TC movements into four difficulty levels using normalized center of pressure (COP) displacement to regulate intensity. Based on each participant’s maximum COP displacement and individual endurance, they assigned three to five movements at a personalized difficulty level. This tailored approach may have contributed to the significant improvements in lower-limb isometric strength observed in their TC group, in contrast to the outcomes in our intervention.

Similarly, significant improvements in MIHS and relative MIHS were observed in favor of the TKD group, whereas no significant changes were found in the TC group. These results are consistent with those reported by Valdes-Badilla et al. [33], who found significant improvements in the non-dominant hand (*p* = 0.001) following a 16-week TKD training intervention in apparently healthy older women. Similarly, Lee et al. [58] reported significant improvements in MIHS of the dominant hand (*p* = 0.03) after 12 weeks of TKD training in older women with stage 2 hypertension, compared to an inactive control group. In this context, the improvements observed in the TKD group may be explained by the striking and blocking techniques, which require sustained isometric strength throughout their execution, particularly through continuous contraction of the hand and forearm flexor muscles [59]. These demands may have led to positive neuromuscular adaptations reflected in MIHS outcomes [41]. In contrast, our findings for TC differ from those reported in a systematic review and meta-analysis by Wehner et al. [60], who found significant improvements (*p* < 0.05) in MIHS in favor of TC interventions in healthy individuals around 60 years of age. Although the authors noted that the increases in MIHS had only a small effect and were below the minimal clinically important difference reported by Bohannon et al. [61], similarly, Elhamrawy et al. [18] reported significant improvements in MIHS (*p* = 0.001) in favor of TC after 12 weeks of training, compared to both an aerobic training group and an inactive control group in older adults post-COVID-19. It is important to note that the meta-analysis by Wehner et al. [60] showed high heterogeneity (99.8%), and the positive results reported by Elhamraw et al. [18] may have been influenced by the negative impact of COVID-19 on physical fitness, which could have made improvements in MIHS through TC more attainable. In contrast, our findings suggest that TC was not a sufficient stimulus to enhance MIHS in apparently healthy older people, likely due to its lower-intensity actions and slow, controlled movements [44].

Regarding the 30 s chair stand test, significant improvements were observed in both the TKD and TC groups, with no significant differences between them. These findings are consistent with those of Kim et al. [62], who reported significant improvements in the 30 s chair stand test (*p* < 0.01) after 12 weeks of TKD intervention in older women with hypertension, compared to an inactive control group. Similarly, Valdés-Badilla et al. [17] found significant improvements (*p* = 0.001) in the 30 s chair stand test following 8 weeks of TKD intervention in healthy older women, compared to an MCT group. These findings are also in line with those reported by Huang et al. [63] in a systematic review and meta-analysis, which found significant improvements (*p* < 0.001) in the 30 s chair stand test in favor of TC interventions among older people with sarcopenia and frailty, compared to both exercise and non-exercise control groups. In this regard, both TKD and TC incorporate techniques that engage the lower limbs. TKD, for example, involves fast-paced movements such as kicking, which require continuous hip flexion and knee flexion–extension [33]. In contrast, TC includes slow, controlled techniques involving shifts in body position and single-leg stances that stimulate motor control and unilateral strength [16]. While both modalities feature constant movement and single-leg support, the key difference lies in the speed of execution [16,62]. Our findings suggest that both TKD and TC interventions were effective in improving performance in the 30 s chair stand test. However, this test is limited to measuring the number of repetitions completed. Therefore, although both groups showed similar outcomes in this measure, we cannot rule out the possibility that TKD, due to the higher velocity of its techniques, may have produced greater gains in other kinetic strength variables, such as power output or rate of force development, compared to TC [41,59].

Finally, for the 30 s arm curl test, significant improvements were observed in the TKD group compared to the TC group. These findings are consistent with those of Valdes-Badilla et al. [17], who reported significant increases in the 30 s arm curl test (*p* < 0.0001) in favor of TKD over MCT in older women after 8 weeks of intervention. Similarly, in a separate study by the same author, significant improvements in the 30 s arm curl test (*p* < 0.001) were found in favor of TKD compared to a control group in apparently healthy older women following a 16-week training program [33]. Our findings differ from those of Elhamraw et al. [18], who reported significant improvements in the 30 s arm curl test (*p* = 0.001) in favor of TC after 12 weeks of training, compared to an inactive control group in older people post-COVID-19. The greater improvements observed in the TKD group compared to the TC group in our study may be attributed to the repeated explosive upper-limb efforts involved in TKD, such as punching [33]. In this context, the accumulated time under tension during TKD techniques may have contributed to enhanced muscle strength endurance by improving forearm muscle efficiency and fatigue tolerance in older women [59]. Conversely, TC may not have provided a sufficient stimulus to improve the 30 s arm curl test in independent older people. The improvements in favor of TC reported by Elhamraw et al. [18] may be explained by the lower baseline physical fitness of participants who had experienced COVID-19, potentially resulting in greater capacity for improvement. However, as few studies have included the arm curl test in TC interventions, further research is warranted to confirm our findings.

### 4.5. Flexibility Performance

Regarding lower-limb flexibility, the TKD group showed significant gains in the sit-and-reach test, whereas no meaningful changes were observed in the TC group. These results are consistent with Valdes-Badilla et al. [33], who also reported improvements after 16 weeks of TKD, and suggest that the dynamic kicking techniques in TKD act as functional flexibility drills, promoting hip range of motion through repeated pre-stretching of the posterior chain [64,65]. In contrast, Niño et al. [66] found long-term benefits of TC on sit-and-reach performance, which may indicate that longer interventions (>16 weeks) are required for TC to elicit measurable adaptations in this outcome.

Conversely, for upper-limb flexibility, the TC group improved significantly in the back scratch test, whereas TKD participants essentially maintained their baseline levels. This aligns with previous evidence showing that TC movements frequently mobilize the shoulder girdle through slow, controlled arcs of motion that involve simultaneous flexion, abduction, and rotation of the arms [60,66]. Such actions may gradually reduce stiffness in periarticular tissues and antagonist muscle coactivation, thereby enhancing shoulder mobility. In contrast, TKD emphasizes explosive strikes and blocking techniques that predominantly require isometric stabilization of the upper limb, which may explain the lack of improvement observed in this group [17,33,42]. Taken together, these results suggest a differential adaptation pattern: TC provides a superior stimulus for upper-limb mobility through repetitive stretching and joint mobilization, whereas TKD appears more effective in enhancing lower-limb flexibility. To expand the benefits of TKD, future programs could integrate specific flexibility drills targeting the upper limbs.

### 4.6. Strengths and Limitations of the Study

Our study presented the following limitations: (i) no analysis of physiological and/or biochemical variables; (ii) no control of food intake to determine the dietary profiles of the participants; (iii) no analysis of the rate of force development during the IMTP to examine the differences in explosive strength between TKD and TC groups; (iv) the final sample size was relatively small (TKD n = 11, TC n = 10), which may have reduced statistical power despite an a priori calculation. Although a post hoc analysis based on VO_2_ at VT1 indicated an achieved power of 77%, this limitation may affect the generalizability of the findings and highlights the need for future studies with larger samples and improved retention strategies; and (v) the absence of follow-up assessments to determine the sustainability of the observed effects over time. Conversely, among the strengths of our study are the following: (i) the comparison of TKD vs. TC with a random assignment of participants, improving the internal consistency of the study; (ii) the use of valid tests frequently used in interventions with older people, improving the external validity of our study; (iii) the inclusion of the IMTP provided a rapid and reliable method to assess maximal isometric strength in older women; (iv) the inclusion of ventilatory threshold-based variables (VT1, VT2, VE/VCO_2_, OUES, COP), which provide a comprehensive and functional view of cardiorespiratory fitness beyond traditional VO_2_ peak; and (v) the use of certified TKD and TC instructors with mastery in exercise for health.

### 4.7. Practical Applications

Adapted TKD demonstrated superior effectiveness in improving cardiorespiratory fitness variables—including VO_2_ VT1, power at VT1, VO_2_/HR VT1, RER VT1, VE/VCO_2_, VO_2_ VT2, power at VT2, VO_2_/HR VT2, COP, and OUES—compared to TC, which only improved power at VT1. Therefore, when the primary goal is to enhance cardiorespiratory fitness in older women, coaches and healthcare professionals may consider prioritizing the prescription of adapted TKD programs. In terms of balance, TKD was more effective than TC, as evidenced by greater improvements in the TUG test. Regarding muscle strength, TKD led to greater improvements in IMTP, relative IMTP, MIHS, relative MIHS, and the 30 s arm curl test. Both TKD and TC were effective in improving lower-limb muscle strength, as shown by similar gains in the 30 s chair stand test. For flexibility, results were mixed: TKD produced improvements in the sit-and-reach test, whereas TC was more effective in the back scratch test. Based on these findings, we recommend implementing adapted TKD programs when the objective is to enhance cardiorespiratory fitness, balance, and muscle strength in independent older women. These programs can be structured as 60-min sessions, three times per week, over a minimum period of 8 weeks. Sessions may include non-contact drills, basic stances, and specific technical components involving upper-limb techniques (e.g., strikes and blocks) and lower-limb movements (e.g., stances, footwork, and kicks), performed individually or in pairs, with or without equipment such as kicking shields or focus pads [15,41]. Importantly, these interventions should be delivered by professionals in physical activity and health who are also certified by the corresponding TKD federation [17,33].

## 5. Conclusions

The adapted TKD intervention proved effective in enhancing cardiorespiratory fitness, balance—as measured by the TUG test—and muscle strength, as evidenced by improvements in IMTP, relative IMTP, MIHS, relative MIHS, and the 30 s arm curl test in independent older women. Regarding flexibility, results were mixed: TKD improved sit-and-reach performance, whereas TC yielded greater gains in the back scratch test. Overall, the TKD program demonstrated superior benefits across multiple domains of physical fitness when compared to TC, positioning it as a promising and comprehensive training strategy for promoting health and functional capacity in older women.

## Figures and Tables

**Table 1:** Participants’ baseline demographic data.

Variables	TKD Group (n = 11)	TC Group (n = 10)	*p*-Value
Age (years)	70.7 ± 7.0	74.8 ± 6.6	0.18
Height (m)	1.58 ± 0.08	1.59 ± 0.06	0.88
Body mass (kg)	64.1 ± 11.6	67.4 ± 8.5	0.47
BMI (kg/m^2^)	25.4 ± 3.9	26.6 ± 3.2	0.44

Data are presented as mean ± standard deviation. TKD: Taekwondo. TC: Tai Chi. *p*-value: statistical significance value. m: meters. kg: kilograms. BMI: body mass index.

## Data Availability

The datasets generated during and/or analyzed during the current research are available from the corresponding author upon reasonable request.

## Supplementary Materials

The following supporting information can be downloaded at https://www.mdpi.com/article/10.3390/life15101511/s1. Table S1 shows the results of the independent samples *t*-test for the initial assessment of cardiorespiratory variables in the Tai Chi and Taekwondo groups. Table S2 presents the results of the independent samples *t*-test for baseline muscle strength variables in the two groups. Table S3 reports the results of the independent samples *t*-test for baseline flexibility variables, while Table S4 displays the results of the independent samples *t*-test for the initial assessment of balance.
