# Supplementary material for: Comparative Effects of Adapted Taekwondo Versus Tai Chi on Health Status in Independent Older Women: A Randomized Controlled Trial
Source: Life (Basel). 2025 Sep 25;15(10):1511. doi: 10.3390/life15101511 (PMC12565102; doi:10.3390/life15101511)
Supplement: Supplementary file 1 [file life-15-01511-s001.zip › life-3890171-supplementary.pdf]

Table S1. Baseline cardiorespiratory variables in the Tai Chi and Taekwondo groups. Data are presented as mean ± standard deviation with 95% confidence intervals. VO_2 = oxygen uptake; HR = heart rate; RER = respiratory exchange ratio; VE/VCO_2 = ventilatory equivalent for carbon dioxide; COP = circulatory power; OUES = oxygen uptake efficiency slope; p: significance value.

Cardiorespiratory variables	Tai Chi (n= 10)	95% CI	Taekwondo (n= 11)	95% CI	p
VO_2 VT1 (mL/kg-min)	11.9 ± 2.1	10.34 – 13.46	13.6 ± 2.8	11.71 – 15.57	0.13
Power VT1 (watts)	41.0 ± 3.1	38.74 – 43.26	41.8 ± 6.0	37.77 – 45.87	0.70
VO_2/HR VT1 (mL/heart beats)	7.6 ± 1.6	6.42 – 8.77	8.8 ± 2.1	7.35 – 10.28	0.16
RER VT1 (VO_2/VCO_2)	0.86 ± 0.03	0.83 – 0.88	0.85 ± 0.03	0.83 – 0.87	0.62
VO_2 VT2 (mL/kg-min)	17.2 ± 2.8	15.15 – 19.25	19.2 ± 3.4	16.98 – 21.56	0.14
Power VT2 (watts)	66.0 ± 12.6	56.95 – 75.05	72.7 ± 13.4	63.67 – 81.79	0.25
VO_2/HR VT2 (mL/heart beats)	9.4 ± 1.5	8.32 – 10.48	10.2 ± 2.9	8.32 – 12.22	0.40
VE/VCO_2 (slope in degrees)	32.3 ± 4.6	28.99 – 35.69	36.4 ± 5.5	32.74 – 40.21	0.08

**COP (average min L)**	27.2 ± 2.9	25.13 – 29.33	28.4 ± 3.1	26.29 – 30.51	0.39
**OUES (mL)**	1.6 ± 0.02	1.59 – 1.63	1.6 ± 0.1	1.56 – 1.70	0.58
**2-minute step test**	96.9 ± 18.2	83.8 – 109.9	105.9± 17.7	94 – 117.8	0.26

Table S2. Baseline muscle strength variables in the Tai Chi and Taekwondo groups. Data are presented as mean ± standard deviation with 95% confidence intervals. IMTP = isometric mid-thigh pull; N = newton; kg = kilogram; MIHS = maximal isometric handgrip strength; kgf = kilogram-force; p: significance value.

Muscle strength variables	Tai Chi (n= 10)	95% CI	Taekwondo (n= 11)	95% CI	p
**IMTP (N)**	1296 ± 156	1184 – 1408	1246 ± 260	1071 - 1421	0.60
**Relative IMTP (N/kg)**	19.4 ± 2.9	17.32 – 21.58	19.5 ± 2.7	17.66 – 21.36	0.96
**MIHS-dominant hand (kgf)**	26.9 ± 5.4	23.03 – 30.77	29.0 ± 7.1	24.27 – 33.91	0.44
**Relative MIHS-dominant hand (kgf/kg)**	0.42 ± 0.12	0.33 – 0.51	0.45 ± 0.09	0.39 – 0.51	0.52
**30-second chair stand**	17.5 ± 4.1	14.56 – 20.44	21.2 ± 4.9	17.97 – 24.58	0.07

**30-second arm curl**	29.5 ± 6.5	24.79 – 34.21	29.7 ± 3.7	27.19 – 32.26	0.92

Table S3. Baseline flexibility variables in the Tai Chi and Taekwondo groups. Data are presented as mean ± standard deviation with 95% confidence intervals. cm = centimeters; p: significance value.

Flexibility variables	Tai Chi (n= 10)	95% CI	Taekwondo (n= 11)	95% CI	p
**Sit-and-reach (cm)**	0.30 ± 12.4	-8.58 – 9.18	3.3 ± 11.3	-4.25 – 10.98	0.56
**Back scratch (cm)**	-11.2 ± 19.6	-25.29 – 2.88	-6.2 ± 11.4	-13.99 – 1.44	0.48

Table S4. Baseline balance variable in the Tai Chi and Taekwondo groups. Data are presented as mean ± standard deviation with 95% confidence intervals. TUG = Timed Up-and-Go; s = seconds; p: significance value.

Balance variable	Tai Chi (n= 10)	95% CI	Taekwondo (n= 11)	95% CI	p
**TUG (s)**	5.9 ± 0.6	5.42 – 6.37	5.1 ± 0.5	4.73 – 5.51	**0.01***
